# Novel angiotensin-converting enzyme and pancreatic lipase oligopeptide inhibitors from fermented rice bran

**DOI:** 10.3389/fnut.2022.1010005

**Published:** 2022-09-15

**Authors:** Jingfei Hu, Huanyu Wang, Nanhai Weng, Tong Wei, Xueqing Tian, Jing Lu, Mingsheng Lyu, Shujun Wang

**Affiliations:** ^1^Jiangsu Key Laboratory of Marine Bioresources and Environment, Jiangsu Ocean University, Lianyungang, China; ^2^Jiangsu Key Laboratory of Marine Biotechnology, Jiangsu Ocean University, Lianyungang, China; ^3^Co-Innovation Center of Jiangsu Marine Bio-industry Technology, Jiangsu Ocean University, Lianyungang, China

**Keywords:** ACE, bioactive oligopeptides, inhibitory kinetic, molecular docking, pancreatic lipase

## Abstract

This study determined the inhibitory activity of oligopeptides against angiotensin-converting enzyme (ACE) and pancreatic lipase through *in vitro* tests, molecular docking, and enzyme inhibition. The results showed that the IC_50_ of GLLGY, HWP, and VYGF for ACE inhibition was 1 mg/mL, and the IC_50_ of HWP for pancreatic lipase was 3.95 mg/mL. Molecular docking revealed that the binding energies between GLLGY, HWP, and VYGF and ACE were –9.0, –8.4, and –9.2 kcal/mol, respectively. The binding free energy between HWP and pancreatic lipase was –7.3 kcal/mol. GLLGY, HWP, and VYGF inhibited ACE compentitively. HWP inhibited pancreatic lipase through non-competition. *in vitro* simulated gastrointestinal digestion, the three oligopeptides still had inhibitory activity and low toxicity. The results revealed that the peptides GLLGY, HWP, and VYGF may be suitable candidates for further research on ACE inhibition, and HWP may be a suitable candidate for studying pancreatic lipase inhibition.

## Introduction

Obesity has recently shown an epidemic trend globally (1). Obesity is also considered the driving force for the development of chronic diseases, such as hypertension, coronary heart disease, and diabetes (2, 3). Therefore, obesity is closely related to lipase activity in humans, and the human body degrades and digests fat taken from food through pancreatic lipase. Then fat was reabsorbed and synthesized in the intestine to lead to fat accumulation (4). The pancreatic lipase inhibitor can effectively inhibit pancreatic lipase activity, impede excessive fat accumulation, and play a role in controlling and treating obesity (5, 6). Orlistat is a drug considered clinically beneficial in treating overweight, but it is associated with side effects such as gastrointestinal (GI) flatulence and arrhythmia (4, 7).

Hypertension is regulated by various mechanisms, one of which is related to the activity of the angiotensin-converting enzyme (ACE) (8). ACE plays a vital role in regulating blood pressure balance by acting on the renin angiotensin–aldosterone system and kallikrein–kinin system (9, 10). ACE inhibitors are widely used in hypertension treatment. However, commonly used ACE inhibitors, such as captopril, enalapril, and benazepril, can cause side effects including renal function damage, phlegm-free dry cough, hyperkalemia, and renal function injury. Safer ACE inhibitors with no side effects are urgently required (8, 11, 12).

At present, most synthetic drugs used in clinics for the prevention and treatment of chronic diseases exhibit side effects, which also urges researchers to focus on natural substances or food for therapeutic uses (13–15). Natural substances and food have many active substances, especially bioactive peptides, which have various human metabolism and physiological regulation functions, easy digestion and absorption, and promote immunity, reduce blood pressure and blood lipid (16, 17). Joyce Irene Boye et al. reported that the IC_50_ of red lentil protease hydrolysate for ACE was 111 ± 1 μmol/L (18). Priti Mudgil et al. found that peptide Leu-Pro exhibits pancreatic lipase inhibitory activity against bovine and camel casein hydrolysates (19). Uriel urbizo Reyes et al. showed that canary seed peptides inhibit ACE and pancreatic lipase. Among them, the peptides LHPQ, QTPHQ, KPVPR, and ELHPQ are noncompetitive inhibitors of ACE. The peptides VPPR, LADR, LSPR, and TVGPR are noncompetitive inhibitors of pancreatic lipase (16). These studies have shown that bioactive peptides can inhibit ACE and pancreatic lipase and can be used to prepare functional foods and nutritional drugs.

In this study, *Bacillus subtilis* MK15 was used to ferment rice bran to extract bioactive peptides and synthesize several better oligopeptides through molecular docking. *in vitro* inhibition experiments, molecular docking, and enzyme inhibition kinetics revealed that these oligopeptides can inhibit ACE and pancreatic lipase. Finally, *in vitro* simulated GI digestion and toxicity studies (*in silico*) were conducted to explore whether these oligopeptides can be used in health products.

## Materials and methods

### Materials

The polypeptide was synthesized by Hangzhou Dangang Biotechnology Co., Ltd (Hangzhou, China). Human ACE, N-[3-(2-furanyl) acryloyl]-l-phenylalanyl glycyl glycine (FAPGG), p-nitrophenyl butyrate (pNPB), and pancreatic lipase were purchased from Sigma Co., St. Louis, Missouri, United States.

### Obtaining bioactive peptides

The peptides used in the study were obtained from *Bacillus subtilis* MK15-fermented rice bran (20). Bioactive peptides were extracted from broth. Ultrafiltration was performed, and DEAE Sepharose Fast Flow ion column and Sephadex G-25 column were used for purification. The peptides identified through LC-MS/MS were FPF, HWP, QSFF, MKNLPKYRQIVHFIKEKIGNG, ALGHIKEAISEGYKVVVVVSAMGR, GLLGY, SHEVK, FSGF, VYGF, QFAKYILFVKDITSKIEEKRG, IANLTEPTDFRIEL RIKRDRG, GLIGY, QSFLQRYYFLFRILP, LFSGF, and PSR. The peptide segments were scored with hydrophobicity, and their inhibitory activities against ACE and pancreatic lipase were measured.

### Determination of angiotensin-converting enzyme inhibitory activity of peptides

According to the experimental method of Yu Fu et al. (8), with slight changes, 50 μL ACE (0.1 U/mL, prepared from borate buffer with pH 8.3 and 80 mM), 50 μL FAPGG (1 mM), and 100 μL borate buffer (80 mM, pH 8.3) were added to a 96-well plate. Then, 100 μL borate buffer was replaced with 1 mg/mL sample, which are three groups of parallel samples, and the initial absorbance values a_1_ and b_1_ were measured at 340 nm. Then, the plate was placed at 37°C and reacted for 30 min. The absorbance values a_2_ and b_2_ were measured at 340 nm (Multiskan GO, Thermo Scientific, Waltham, MA, USA). The absorbance value was incorporated into the following formula to calculate the inhibition rate of polypeptide against ACE activity:


(1) $$\mathrm{ACE}\mathrm{in}\text{h}\mathrm{ibition}\mathrm{rate}\;\left(\%\right)=\frac{\left(A-B\right)}{A}\times 100$$

Where a_1_ and b_1_ are initial absorbance values of the blank and sample groups, respectively. a_2_ and b_2_ are absorbance values of the blank and sample groups after the reaction, respectively. A = a_1_–a_2_; B = b_1_–b_2_.

### Determination of pancreatic lipase inhibitory activity of peptides

According to the method of Magdalena mendoza-s á nchez (1). In the blank group, 100 μL of 0.1 M sodium phosphate buffer (pH 7.2), 50 μL pNPB (5 mM), and 50 μL pancreatic lipase (0.2 U/mL) were added to a 96-well plate. In the blank control group, 150 μL sodium phosphate buffer and 50 μL pNPB were added to the 96-well plate. In the sample group, 50 μL sodium phosphate buffer, 50 μL pNPB, 50 μL sample (1 mg/mL), and 50 μL pancreatic lipase were added to the 96-well plate. For the sample blank group, 100 μL sodium phosphate buffer, 50 μL pNPB, and 50 μL sample were added to the 96-well plate. At 37°C, the absorbance values A, B, C, and D were measured at 405 nm after 30 min of reaction. The absorbance values were incorporated into the following formula to calculate the inhibition rate of polypeptide against pancreatic lipase activity:


(2) $$\mathrm{Pancreatic}\mathrm{lipase}\mathrm{inhibition}\mathrm{rate}\;\left(\%\right)=\left(1-\frac{C-D}{A-B}\right)\times 100$$

Where A, B, C, and D are absorbance values of the blank, blank control, sample, and sample blank groups, respectively.

### Molecular docking

Autodock Vina 1.1.2 docking software was used for molecular docking. From the protein database^1^, the crystal structures of human ACE (PDB ID:1O86; resolution 2.00 Å) and porcine pancreatic lipase (PDB ID:1ETH; resolution 2.80 Å) were retrieved in PDB format. Subsequently, co-crystalline ligands and water molecules were removed from the protein structures using PyMOL version 2.5.0.

When the polypeptide was docked with ACE, the docking site was the same as that reported by Dong (21). The grid size (xyz point) was set to 126, 126, and 126, and the specified size (x, y, and z) of the grid center was 43.82, 38.31, and 46.65. Other parameters were set to default values. When the polypeptide was docked with porcine pancreatic lipase, the docking site of Akpovwehwee A. Anigbor et al. (4). The grid size (xyz point) was set to 126, 126, and 126, and the specified size (x, y, and z) of the grid center was 64.00, 29.16, and 125.43. Other parameters were set to default values. After docking, the best objects were elected and analyzed using PyMOL 2.5.0.

### Inhibitory kinetic analysis

The inhibition kinetics of oligopeptides GLLGY, HWP, and VYGF on ACE and pancreatic lipase were tested to determine their efficiencies. For the ACE inhibition test, the concentration of the samples GLLGY, HWP, and VYGF was 1 mg/mL, and the concentrations of the substrate FAPGG were 0.2, 0.4, 0.6, 0.8, and 1 mM. For the pancreatic lipase inhibition test, the sample HWP concentration was 1 mg/mL and the substrate pNPB concentrations were 1, 2, 3, and 5 mM. The initial velocity data were used to construct the Lineweaver–Burke plots to determine the enzyme Km (Michaelis constant), Vmax (maximum velocity), and Ki (inhibitory binding constant) (8, 22).

### *In vitro* simulated gastrointestinal digestion

Simulated GI digestion through an *in vitro* pepsin–pancreatin hydrolysis method was carried out (23). The oligopeptides GLLGY, HWP, and VYGF were re-dissolved (3% w/v in distilled water) and adjusted to pH 2.0 with 1 M HCl. Then, pepsin (4% weight as received/weight of protein in the powder) was added. The mixture was incubated at 37°C for 2 h. The pH was then adjusted to 5.3 with 0.9 M NaHCO_3_ solution and further to pH 7.5 with 1 M NaOH. Pancreatin was added (4% weight as received/weight of protein in the powder), and the mixture was further incubated at 37°C for 2 h. To terminate the digestion, the test tubes were kept in boiling water for 10 min. The gastric digests (GDs) and gastric-intestinal digests (GIDs) were obtained through centrifugation (8000 × *g*, 10 min, 4°C), and then, the supernatants were collected, lyophilized, sealed in plastic bags, and stored at 4°C until further analysis. The ACE and pancreatic lipase inhibitory activities of GDS and GID were measured at a concentration of 1 mg/mL, and the results were expressed as activity (%) relative to that without any treatment (control group, 100%). All determinations were made in triplicate.

### Toxicity studies (*in silico*)

The evaluation of pharmacologically active substances during the new drug development is an indispensable parameter. The *in vitro* evaluation of ADMET (absorption, distribution, metabolism, excretion, and toxicity) provides data for the identification of new molecules (24). ADMET parameters were calculated using the free web interface pkCSM^2^ for predicting the toxicities of the oligopeptides GLLGY, HWP, and VYGF (25).

### Statistical analysis

Three parallel tests were conducted in all experiments. Data are presented as mean ± SD. SPSS version 17.0 for Windows was used for statistical analysis. *P* < 0.05 was set as the threshold for statistical significance.

## Results

### Inhibitory activity *in vitro*

#### ACE inhibitory activity *in vitro*

**FIGURE 1:**
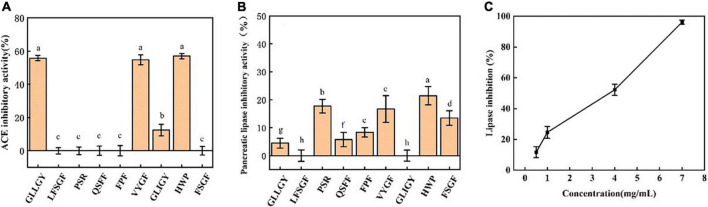
**(A)** The ACE inhibitory activity of peptides. The pancreatic lipase inhibitory activities of **(B)** peptides and **(C)** HWP. The small letters represent the significant difference *p* < 0.05.

Nine peptides were selected for the ACE inhibitory activity assay. As shown in Figure 1A, at 1 mg/mL, the ACE inhibitory activities of GLLGY, VYGF, and HWP were considerably higher than those of other peptides. The IC_50_ of GLLGY, VYGF, and HWP for ACE was 1 mg/mL.

#### Pancreatic lipase inhibitory activity *in vitro*

As shown in Figure 1B, at 1 mg/mL, HWP exhibited the highest inhibitory activity against pancreatic lipase (inhibition rate: 20%), followed by PSR. A slight difference was observed in the inhibitory activities of VYGF and PSR against pancreatic lipase, and the inhibition rate of FSGF was 13%. The pancreatic lipase inhibition rate of the remaining peptides was <10%. Next, HWP was subjected to a gradient test of concentration and inhibitory activity. A linear relationship was observed between the lipase inhibitory activity of HWP and HWP concentration. With an increase in the concentration, the inhibitory activity also increased, and the IC_50_ of HWP for pancreatic lipase was 3.95 mg/mL (Figure 1C).

### Molecular docking

#### Molecular docking of angiotensin-converting enzyme

**FIGURE 2:**
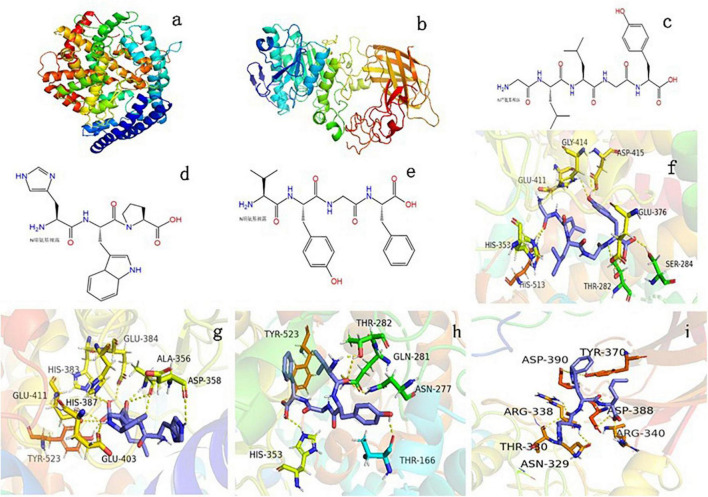
Binding of oligopeptides to human ACE and porcine pancreatic lipase. Three-dimensional structures of **(a)** human ACE and **(b)** porcine pancreatic lipase. Structural diagrams of **(c)** GLLGY peptide, **(d)** HWP peptide, and **(e)** VYGF peptide. A visual interaction diagram of **(f)** GLLGY, **(g)** HWP, and **(h)** VYGF with ACE. **(i)** A visual interaction diagram of HWP with pancreatic lipase. The yellow dotted lines indicate hydrogen bonds.

The GLLGY characteristics are as follows: isoelectric point, 6.2; average hydrophilicity, --1.1; hydrophilic residue, 0%; net charge, 0.0 at pH = 7.0. The HWP characteristics are as follows: isoelectric point, 7.8; average hydrophilicity, --1.3; hydrophilic residue, 0%; net charge, 0.1 at pH = 7.0. The VYGF characteristics are as follows: isoelectric point, 6.2; average hydrophilicity, --1.5; hydrophilic residue, 0%; net charge, 0.0 at pH = 7.0^3^. Figure 2a depicts the three-dimensional structure of human ACE (PDB ID:1O86). The structural diagrams of GLLGY, HWP, and VYGF are presented in Figures 2c,d,e, respectively. GLLGY was bound to 9 ACE residues to form hydrogen bonds: Thr282, 2.2 Å; Ser284, 2.2 Å; His353, 2.3 Å; Glu376, 2.2 Å; Glu411, 2.5 Å; Gly414, 2.7 Å; Asp415, 2.1 Å and 2.2 Å; and His513, 2.2 Å (Figure 2f, hydrogen bond distance not marked). HWP was bound to 11 ACE residues to form hydrogen bonds: Ala356, 1.9 Å, 2.1 Å, and 3.4 Å; Asp358, 2.6 Å; His383, 2.6 Å; Glu384, 2.4 Å; His387, 2.4 Å; Glu403, 2.6 Å; Glu411, 3.3 Å and 3.4 Å; and Tyr523, 1.8 Å (Figure 2g, hydrogen bond distance not marked). VYGF was bound to 6 ACE residues to form hydrogen bonds: Thr166, 2.0 Å; Asn277, 2.5 Å; Gln281, 2.7 Å; Thr282, 2.1 Å; His353, 2.4 Å; and Tyr523, 2.3 Å (Figure 2h, hydrogen bond distance not marked). The binding free energies of GLLGY, HWP, and VYGF were –9.0, –8.4, and –9.2 kcal/mol, respectively.

#### Molecular docking of pancreatic lipase

Figure 2b depicts the three-dimensional structure of PDB ID:1ETH. Molecular docking was used to simulate the interaction between HWP and porcine pancreatic lipase. The binding free energy of HWP was –7.3 kcal/mol. HWP was bound to the bottom of the pocket and had hydrogen bond interactions with 9 residues of pancreatic lipase: Asn329, 2.3 Å; Thr330, 3.5 Å; Arg338, 2.5Å; Arg340, 2.5 Å; Tyr370, 2.8 Å; Asp388, 1.9 Å, 2.5 Å, and 3.5 Å; and Asp390, 2.6 Å (Figure 2i, hydrogen bond distance not marked).

### Enzyme inhibition kinetics

#### Kinetics of angiotensin-converting enzyme inhibition

**FIGURE 3:**
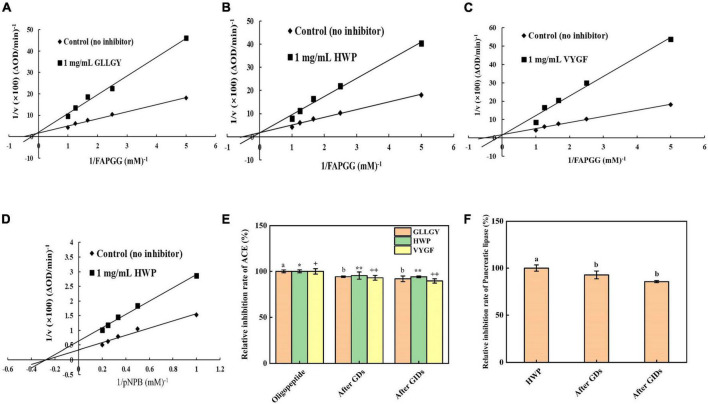
Lineweaver–Burk plots of ACE-catalyzed reactions in the presence of **(A)** GLLGY, **(B)** HWP, and **(C)** VYGF. Lineweaver–Burk plots of pancreatic lipase-catalyzed reactions in the presence of **(D)** HWP. Each dot represents a mean of three independent experiments conducted in triplicates. Effect of simulated GI digestion on **(E)** ACE activity and **(F)** pancreatic lipase activity. The small letters represent the significant difference *p* < 0.05.

Enzyme kinetic studies showed that GLLGY, HWP, and VYGF were competitive inhibitors of ACE (Figures 3A,B,C). Vmax remained unchanged and Km increased. The calculated inhibitory binding constants (Ki) of GLLGY, HWP, and VYGF were 1.573, 1.340, and 0.933 mM, respectively. Ki is an indicator that defines the binding (affinity) ability of inhibitors to enzymes to form enzyme–inhibitor complexes, and a lower Ki value indicates a higher affinity. GLLGY, HWP, and VYGF compete with the substrate for the ACE active center and form a reversible enzyme–inhibitor complex with ACE, thereby preventing the substrate from binding to the enzyme (16).

#### Kinetics of pancreatic lipase inhibition

Enzyme kinetic studies revealed that HWP was a non-competitive inhibitor of pancreatic lipase (Figure 3D). Vmax decreased and Km remained unchanged. The calculated inhibitory binding constant (Ki) of HWP was 2.583 mM. pNPB and HWP bind at different sites of pancreatic lipase, which reduces the pancreatic lipase activity (26).

### Oligopeptides simulated gastrointestinal digestive activity

#### Effect of simulated gastrointestinal digestion on angiotensin-converting enzyme activity

As shown in Figure 3E, the inhibition rate of GLLGY against ACE without GI digestion was 55.800%, after gastric digestion was 52.570%, and after GI digestion was reduced to 51.271%. The inhibition rate of HWP against ACE without GI digestion was 57.020%, after gastric digestion was 54.391%, and after GI digestion was reduced to 53.631%. The inhibition rate of VYGF against ACE without GI digestion was 54.757%, after gastric digestion was 50.922%, and after GI digestion was reduced to 49.064%. After *in vitro* simulation of GI digestion, GLLGY, HWP, and VYGF decreased the ACE activity slightly. The inhibitory effect of GDS and GIDS on ACE did not change significantly, and the oligopeptides GLLGY, HWP, and VYGF continued to inhibit ACE after GI digestion.

#### Effect of simulated GI digestion on pancreatic lipase activity

As shown in Figure 3F, the inhibition rate of HWP against pancreatic lipase without GI digestion was 21.451%, after gastric digestion was 19.887%, and after GI digestion was reduced to 18.353%. After *in vitro* GI simulation, HWP decreased the pancreatic lipase activity lightly. The inhibitory effect of GDS and GIDS on pancreatic lipase did not change significantly, and the oligopeptide GLLGY continued to inhibit pancreatic lipase after GI digestion.

### Toxicity studies (*in silico*) of oligopeptides

On the pkCSM website, AMES toxicity shows that the three oligopeptides do not cause cancer and are not hERG I/II inhibitors. The maximum tolerated dose of ≤ 0.477 log (mg/kg/day) is considered low and that of >0.477 log (mg/kg/day) is considered high. The dose of HWP was considered low, whereas that of GLLGY and VYGF was considered high. *Tetrahymena pyriformis* is a protozoan bacterium whose toxicity is often used as a toxic endpoint. The *T. pyriformis* toxicity value of > –0.5 log μg/L is considered toxic. The toxicity of the oligopeptides GLLGY, HWP, and VYGF was > –0.5 log μg/L. The lethal concentration (LC50) value represents the concentration of a molecule necessary to cause the death of 50% of the fathead minnows. LC50 values of <0.5 mM (log LC50 <-0.3) represent high acute toxicity. The LC50 values of GLLGY, HWP, and VYGF were >0.5 mM. The three oligopeptides were regarded as of low toxicity because the toxicity was likely associated with the disrupted function of the liver. These oligopeptides did not cause skin sensitization. Thus, these three oligopeptides were concluded to be of low toxicity (Table 1).

**TABLE 1:** Toxicity studies (*in-silico*) of oligopeptides GLLGY, HWP, and VYGF.

Descriptors	GLLGY predicted value	HWP predicted value	VYGF predicted value	Unit
AMES toxicity	No	No	No	Categorical (Yes/No)
Max. tolerated dose (human)	0.662	0.138	0.552	Numeric (log mg/kg/day)
hERG I inhibitor	No	No	No	Categorical (Yes/No)
hERG II inhibitor	No	No	No	Categorical (Yes/No)
Oral Rat Acute Toxicity (LD50)	2.308	1.969	2.612	Numeric (mol/kg)
Oral Rat Chronic Toxicity (LOAEL)	3.726	2.451	3.776	Numeric (log mg/kg_bw/day)
Hepatotoxicity	Yes	Yes	Yes	Categorical (Yes/No)
Skin Sensitisation	No	No	No	Categorical (Yes/No)
*T. Pyriformis* toxicity	0.285	0.285	0.285	Numeric (log ug/L)
Minnow toxicity	5.934	4	5.464	Numeric (log mM)

## Discussion

According to the report of Dong Wei et al. (21), the ACE (PDB ID: 1O86) active site is mainly composed of three active pockets and Zn^2+^, which contain Ala354, Glu384, Tyr523, Gln281, His353, Lys511, His513, Tyr520, and Glu162 residues. GLLGY, HWP, and VYGF are docked with ACE. GLLGY formed 9 hydrogen bonds with the ACE residues Thr282, Ser284, His353, Glu376, Glu411, Gly414, Asp415, and His513 to produce 9 hydrogen bonds (Figure 2f). HWP formed 11 hydrogen bonds with the ACE residues Ala356, Asp358, Glu384, His383, His387, Glu403, Glu411, and Tyr523 (Figure 2g). VYGF formed 6 hydrogen bonds with the ACE residues Thr166, Asn277, Thr282, Gln281, His353, and Tyr523 (Figure 2h).

Dong Wei et al. found that the peptide PR inhibited ACE activity in a competitive manner and formed 6 hydrogen bonds with the ACE residues Asp415, Glu383, Glu384, Lys511, Tyr520, and Gln281 (21). Mingyang Li et al. reported that RGLSK formed 10 hydrogen bonds with the ACE residues Ala354, Glu384, Tyr523, Glu376, Asp415, His383, and Glu411 (27). Compared with the docking of these peptides with ACE, it can be found that they bind to S1 (Ala354, Glu384, and Tyr523) and S2 (Ala354, Tyr523, and His353) active pockets and inactive sites like these peptides. WG binds to the ACE residues Arg157, Thr7, and Glu17 and does not bind to the ACE active sites; therefore, it has a low inhibitory effect on ACE (8). In docking, hydrogen bonds play a critical role in stabilizing complex ligands and receptors. Hydrogen bonds formed enhance the interaction between the polypeptide and ACE, thus leading to strong ACE inhibition (28). According to the enzyme inhibition kinetics experiment, GLLGY, HWP and VYGF were competitive inhibitors (Figures 3A,B,C) that competed with the substrate and combined with the enzyme active site to inhibit ACE activity, as observed through molecular docking (Figure 2). GLLGY, HWP, and VYGF each contain two hydrophobic amino acids, and the strong inhibition by the polypeptide may be related to the presence of these hydrophobic amino acids (29).

The main active sites of pancreatic lipase (PDB ID: 1ETH) are Ser153, Asp177, His264, Phe78, Ile79, His152, Phe216, Trp253, and Arg257 (19). Sha Li et al. found that apigenin interacted with the active center residues of pancreatic lipase and competitively inhibited its activity, with an IC_50_ value of 0.45 ± 0.03 mM (22). HWP interacted with the pancreatic lipase residues Asn329, Thr330, Arg338, Arg340, Tyr370, Asp388, and Asp390 to form 9 hydrogen bonds (Figure 2i); however, these residues are not the active sites of pancreatic lipase. According to the enzyme inhibition kinetics test, HWP is a non-competitive inhibitor (Figure 3D), which may be the reason for its low inhibition rate. The IC_50_ value of HWP was 8.988 mM (Figure 1C), which is far lower than the IC_50_ of apigenin. However, HWP has two hydrophobic amino acids tryptophan and proline, and non-polar residues play a crucial role in establishing the interaction with lipophilic enzymes. Thus, this may also be the reason for HWP’s high rate of pancreatic lipase inhibition (8).

The inhibitory activities of oligopeptides GLLGY, HWP, and VYGF after simulated GI digestion *in vitro* were not considerably lower than those before digestion. Tausif Ahmed (30) described the structural characteristics of bioactive peptides and their stability in simulated GI digestion. Their research showed that peptides resistant to *in vitro* GI digestion have a shorter chain length, smaller molecular weight (23), lower hydrophobicity, and higher positive net charge at pH 7.0. The average chain length and molecular weight of peptides resistant to *in vitro* GI digestion were 4.5 ± 2.0 amino acid residues and 547.78 ± 233.17 g/mol, respectively, and their net charges were slightly positive. Peptides with a lower molecular weight may possess fewer protease recognition and cleavage sites (30). The oligopeptide GLLGY contains 5 amino acids, with a molecular weight of 522.292 g/mol and net charge of 0.0 at pH = 7.0. The oligopeptide HWP contains 3 amino acids, with a molecular weight of 439.209 g/mol and net charge of 0.1 at pH = 7.0. The oligopeptide VYGF contains 4 amino acids, with a molecular weight of 485.241 g/mol and net charge of 0.0 at pH = 7.0, which is similar to that of the reported stable peptide. This may be the reason the oligopeptides GLLGY, HWP, and VYGF continued to exhibit strong inhibitory activity in the simulated GI digestion *in vitro* (Figures 3E,F). The three oligopeptides were predicted to be low toxicity by the pkCSM website (Table 1).

Obesity is accompanied by chronic diseases such as hypertension, hyperglycemia and coronary heart disease, which affect human health (3). In the metabolism of dietary fat, pancreatic lipase plays a major role to promote fat absorption of small intestine, which leads to fat accumulates and obesity. Therefore, it is an important recognized target for controlling obesity (31). ACE converts the inactive protein angiotensin I into an effective vasoconstrictor angiotensin II, which leads to an increase in blood pressure (12). Myocardial infarction and stroke that are significantly relative with higher blood pressure are top mortality in the world (32). Hence, the inhibitors of ACE and pancreatic lipase are very important to protect human health and has been widely concerned by researchers.

Study showed that oligopeptides could inhibit pancreatic lipase and ACE *in vitro*, and they had the same effect *in vivo*. Luis Jorge coronado-c á Ceres *et.al* studied the inhibitory effect of cocoa protein (CP) hydrolysate (CPH) on pancreatic lipase, and found that cocoa peptides EEQR, GGER QTGVQ and VSTDVNIE had inhibitory effect on pancreatic lipase. The IC_50_ of CP hydrolysate was 1.38 mg/mL. *In vivo* experiments, compared with the high-fat diet group, the high-fat diet and CP group significantly reduced the apparent absorption rate of fat (33). Li Peng et.al. researched on the inhibitory effect of pistachio hydrolysate on ACE and found that the IC_50_ of oligopeptide ACKEP on ACE was 126 μM. Four hours after oral administration in mice, the pepsin–trypsin hydrolysate (Pe–Tr–H) can reduce systolic blood pressure (SBP) by about 22 mmHg and diastolic blood pressure (DBP) by about 16 mmHg *in vivo* (34). Our oligopeptides had the ability to inhibit ACE and pancreatic lipase *in vitro*, furthermore, they had anti digestion ability. It is preliminarily inferred that they might have an inhibitory effect *in vivo* and promote human health. The oligopeptides extracted from fermented rice bran are worthy to be investigated the effect *in vivo* and application in further.

## Conclusion

The biological activity of better peptide components was determined *in vitro*. The IC_50_ of GLLGY, HWP, and VYGF for ACE inhibition was 1 mg/mL and that of HWP for pancreatic lipase was 3.95 mg/mL. Molecular docking results showed that 9, 11, and 6 hydrogen bonds were present between GLLGY, HWP, and VYGF and ACE, respectively. Nine hydrogen bonds were present between HWP and pancreatic lipase. GLLGY, HWP, and VYGF competitively inhibited ACE, and HWP non-competitively inhibited pancreatic lipase. The oligopeptides GLLGY, HWP, and VYGF continued to exhibit strong inhibitory activity during simulated GI digestive activity. Three oligopeptides are low toxicity. The oligopeptides extracted from fermented rice bran have great prospective application for protect human health.

## Data availability statement

The original contributions presented in this study are included in the article/supplementary material, further inquiries can be directed to the corresponding authors.

## Author contributions

JH: conceptualization and writing—original draft preparation. HW: data curation and resources. NW: visualization and investigation. TW: supervision. XT: methodology. JL: software and validation. ML: writing—review and editing. SW: funding acquisition and project administration. All authors contributed to the article and approved the submitted version.

## Footnotes

1
https://www.rcsb.org/

2
http://structure.bioc.cam.ac.uk/pkcsm

3
https://pepcalc.com/
